# Effect of Surface Interactions on Microsphere Loading in Dissolving Microneedle Patches

**DOI:** 10.1021/acsami.2c05795

**Published:** 2022-06-22

**Authors:** Derek Jang, Jie Tang, Steven P. Schwendeman, Mark R. Prausnitz

**Affiliations:** † Wallace H. Coulter Department of Biomedical Engineering at Georgia Tech and Emory University, Georgia Institute of Technology, Atlanta, Georgia 30332, United States; ‡ Department of Pharmaceutical Sciences and the Biointerfaces Institute, University of Michigan, Ann Arbor, Michigan 48109, United States; § School of Chemical & Biomolecular Engineering, Georgia Institute of Technology, Atlanta, Georgia 30332, United States

**Keywords:** microneedle patch, microsphere loading, surface interactions, hydrophobicity, electrostatic repulsion, drug delivery

## Abstract

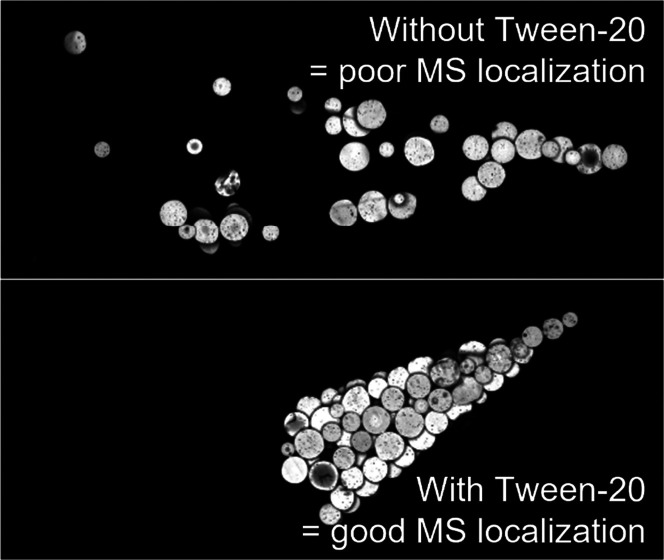

Microneedle (MN) patches enable simple self-administration of drugs via the skin. In this study, we sought to deliver drug-loaded microspheres (MSs) using MN patches and found that the poly(lactic-*co*-glycolic acid) (PLGA) MSs failed to localize in the MN tips during fabrication, thereby decreasing their delivered dose and delivery efficiency into skin. We determined that surface interactions between the hydrophobic MSs and the poly(dimethylsiloxane) (PDMS) mold caused MSs to adhere to the mold surface during casting in aqueous formulations, with hydrophobic interactions largely responsible for adhesion. Further studies with polystyrene MSs that similarly carry a negative charge like the PLGA MSs demonstrated both repulsive electrostatic interactions as well as adhesive hydrophobic interactions. Reducing hydrophobic interactions by addition of a surfactant or modifying mold surface properties increased MS loading into MN tips and delivery into porcine skin ex vivo by 3-fold. We conclude that surface interactions affect the loading of hydrophobic MSs into MN patches during aqueous fabrication procedures and that their modulation with the surfactant can increase loading and delivery efficiency.

## 1 Introduction

Drugs are administered in such a way as to achieve plasma concentrations within a therapeutic window, with a lower bound of the minimum efficacious concentration and an upper bound of the minimum toxic concentration.^1^ Maintaining drug concentration within that window with conventional dosage forms (oral, bolus injections) is difficult, especially in the case of chronic conditions, where frequent dosing can reduce patient compliance.^2^ An ideal controlled release drug delivery system therefore aims to maintain drug concentration within the therapeutic window for increased efficacy and reduced side effects, improve patient compliance by reducing the number of doses, and enable clearance or biodegradation in the body.^3,4^

Controlled rerlease drug delivery systems made of biodegradable materials such as poly(lactic acid) (PLA) and poly(lactic-*co*-glycolic acid) (PLGA) can be engineered for tailored release,^5−7^ and much work using these polymers has involved developing microspheres (MSs) that are injected after suspension in an injection vehicle. However, these MS formulations usually require administration by hypodermic injection, which often requires professional healthcare personnel and proper sharps disposal and can reduce patient compliance.^8^

Microneedle (MN) patches present an attractive, minimally invasive, self-administrable drug delivery platform as an alternative to hypodermic injection.^9−11^ MNs are tapered structures that measure hundreds of microns in height with a small (usually under 10 μm) tip radius. This geometry allows MNs to puncture the stratum corneum barrier layer of the skin, which is about 10–20 μm thick,^12^ thereby minimizing contact with blood vessels and nerves in the dermis.^13,14^ Originally engineered as solid structures that create microchannels in the skin for the delivery of skin-impermeable drugs, MNs have more recently been designed to directly deliver drugs into skin by being coated onto the MN surface,^13−16^ infused through hollow channels within the MNs, or by being incorporated into water-soluble MNs that dissolve in the skin to deliver the encapsulated drug.^13−16^

Dissolving MNs have mainly been investigated for bolus delivery of therapeutics and vaccines due to their fully water-soluble designs, but recent work has involved engineering MNs for controlled release over time. One way is to fabricate MNs out of biodegradable polymers for extended-release.^17−22^ Another way is to integrate well-characterized controlled release MSs made of hydrophobic biodegradable materials with dissolving MNs.^23−27^ In this study, we followed this latter approach by incorporating etonogestrel (ENG)-loaded PLGA MSs into dissolving MN patches for the development of a self-administrable, long-acting contraceptive patch.

We are motivated to develop a MS-loaded MN patch for extended delivery of ENG to address an unmet contraceptive need. Especially in developing countries, women may lack access to contraceptive services or may not find contraceptive options acceptable due to perceived safety or side effects, personal or family opposition to contraception, belief that pregnancy is unlikely, or provider bias.^28^

Longer-acting contraceptives such as implants and injectables have seen expanded use in regions with a large unmet need,^29^ but these contraceptives require administration by trained healthcare providers or proper training for self-administration in the case of the subcutaneous injectable, Sayana Press.^30^ We believe a self-administrable MN patch loaded with controlled-release MSs may better address this large unmet need as a method that requires no expertise to use.

A key consideration in the development of these MN patches was optimizing for the amount and efficiency of drug delivery due to the small size of the MN patch and the relatively large drug (and polymer) dose needed for months-long contraception. To fabricate a dissolving MN patch, a water-soluble formulation is typically cast in an aqueous solution onto a MN patch mold by a variety of methods, such as degassing by vacuum oven,^31^ centrifugation,^32^ or negative pressure with a vacuum chuck.^33^ Once cast, the MNs solidify either by drying (at room, desiccated, or heated conditions) or possibly by polymerization,^31^ after which the MN patch can be demolded and stored or additionally dried.

For efficient delivery, the payload should be localized to the MN tips, where they can be inserted and deposited deep into skin. When fabricating MS-loaded MN patches, we found that MSs can fail to localize to the MN tips therefore reducing delivery of the MSs into skin.

Our study aimed to identify the cause of the poor localization of MSs in the MN tips, understand the mechanism involved, and develop interventions to improve MS localization to the MN tips. We first found that the poor localization was due to MS adhesion to the MN mold walls during fabrication. We investigated potential surface interaction mechanisms responsible for the adhesion and found that hydrophobic interactions played a major role in the adhesion while electrostatic interactions in our studied systems were repulsive. Finally, we developed interventions to adhesive hydrophobic interactions and improved MS loading and localization in MNs and delivery to the skin.

## 2 Results and Discussion

### 2.1 Poor Localization of ENG-MSs in MN Tips

Our first objective was to assess the localization of MSs in MN tips during MN patch fabrication using MSs made of 73% w/w PLGA and 27% w/w etonogestrel (ENG-MS) cast as an aqueous suspension onto a MN mold made of poly(dimethylsiloxane) (PDMS). Our goal is to maximize the MSs localized to the MN tip, since these MSs should be delivered into skin by the MNs, whereas MSs located away from the tip will likely remain in the patch after skin application.

**Figure 1:**
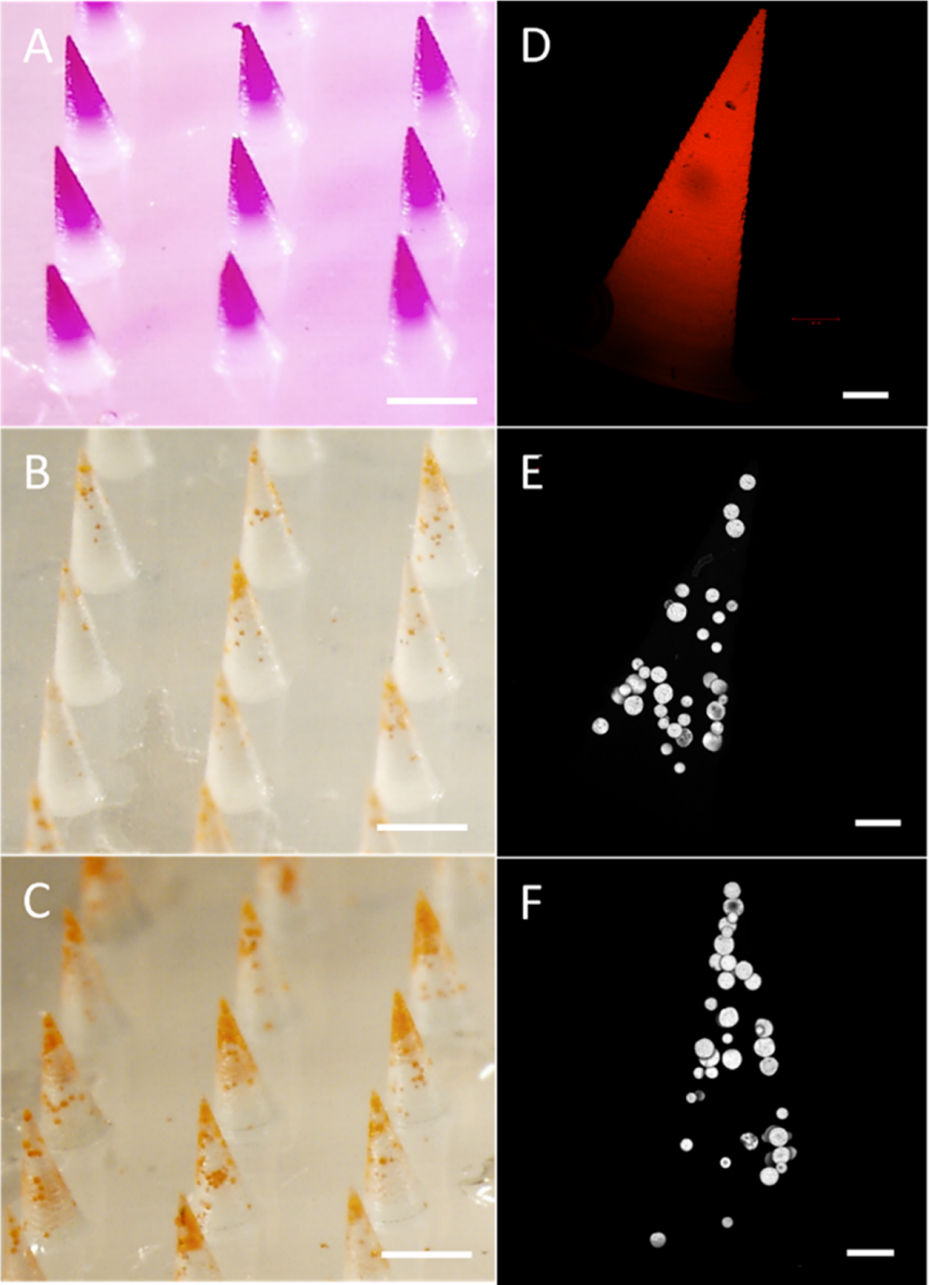
Localization of sulforhodamine B and ENG-MSs in MN tips. Representative brightfield microscopy images of sections of MN patches prepared with a first-cast solution containing (A) sulforhodamine B cast with vacuum chuck, (B) ENG-MSs cast with vacuum chuck, and (C) ENG-MSs cast with centrifugation. Scale bars are 500 μm. Representative confocal microscopy images of individual MNs prepared with a first-cast solution containing (D) sulforhodamine B cast with vacuum chuck, (E) ENG-MSs cast with vacuum chuck, and (F) ENG-MSs cast with centrifugation. The MN casting solution comprised 6% w/v PVP + 6% w/v sucrose in water with 0.1% w/v (A) sulforhodamine B or (B) ENG-MSs. The backing casting solution comprised 18% w/v PVP + 18% w/v sucrose in water. ENG-MSs had a diameter of 37 μm. Scale bars are 100 μm.

As a starting point, we cast an aqueous solution of sulforhodamine B onto a MN mold and found that this casting of a soluble marker compound successfully localized it in the MN tips (Figure 1A,D), consistent with prior findings.^31,32^ This observation indicates that the vacuum applied during casting effectively drew the casting solution into the MN mold cavities and that the dissolved sulforhodamine B localized where the casting solution flowed in the mold. In contrast, when following a similar protocol with a casting solution suspending ENG-MSs, we found that the ENG-MSs failed to evenly localize in the MN mold tips (Figure 1B,E).

**Figure 2:**
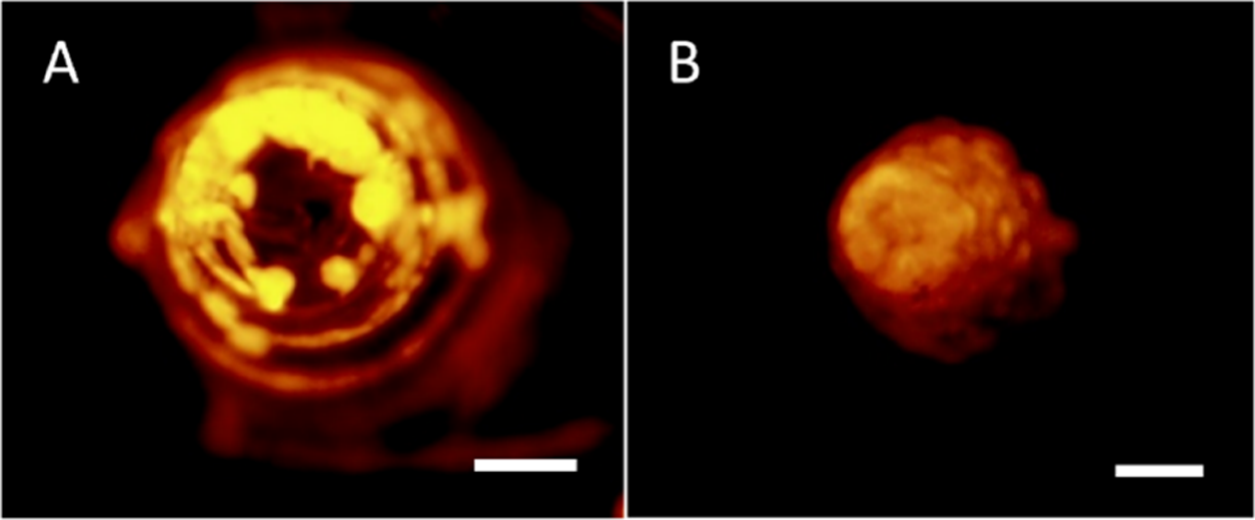
Representative fluorescence microscopy images of a MN loaded with fluorescently labeled MSs. The MN was cut radially at various axial positions and imaged from top-down. Cuts were made outside the packed MN tip at (A) ∼540 μm and inside the packed tip at (B) ∼360 μm. The casting solution comprised 6% w/v PVP + 6% w/v sucrose in water with 0.1% w/v rhodamine 6G-labeled PLGA MSs. The backing solution comprised 18% w/v PVP + 18% w/v sucrose in water. ENG-MSs had a diameter of 37 μm. Scale bars are 100 μm.

To more effectively drive the ENG-MSs into the MN tips, we filled the mold under centrifugation with a *g*-force of 3000*g*. This helped push the ENG-MSs further down into the MN tips (Figure 1C,F) but was still insufficient to fully localize the MSs in the tips. We hypothesized that the poor localization of ENG-MSs in the MN tips was due to their adherence to the PDMS mold walls during the casting process. To test this hypothesis, we cut ENG-MS-loaded MNs at different heights to image ENG-MS localization as a function of position in the MN (Figure 2). This analysis showed that at locations in the MNs outside the tip, the ENG-MSs were localized along the circumference of the MN in a monolayer (Figure 2A), whereas the ENG-MSs within the tip were densely packed, filling the interior of the mold (Figure 2B). This indicated that ENG-MSs that failed to localize to the MN tip were adhering to the mold walls and suggested that we should investigate interventions to influence surface forces between ENG-MSs and the mold walls to increase MN tip loading.

### 2.2 Effect of Hydrophobicity on MS Localization

Guided by these observations, we further hypothesized that MS adhesion to the mold walls is primarily mediated by hydrophobic interactions between MSs and mold surfaces because PLGA, ENG, and PDMS are all hydrophobic and casting was carried out as an aqueous suspension of MSs. We further focused on hydrophobic forces because they are generally the strongest and longest-ranged of the three noncovalent interactions (i.e., hydrophobic, electrostatic, and van der Waals forces^34^).

Additionally, both PLGA^35,36^ and PDMS^37,38^ are negatively charged due to carboxyl groups and Si–O bonds, respectively, suggesting that any electrostatic interactions would be repulsive (as we further investigated below). We did not specifically study van der Waals forces because these forces are generally much smaller than the other two forces.^34^ Finally, we expected that possible MS–MS interactions played a smaller role because the monolayer of MSs on the mold wall suggested that MS-wall interactions were most important.

#### 2.2.1 Hydrophobicity Determined by Water Contact Angle Measurements

We used three approaches to modulate hydrophobic interactions between MSs and the mold walls, with the objective of weakening those interactions to promote MS localization in MN tips. First, we decreased the mold hydrophobicity by incorporating up to 1% w/w PDMS-poly(ethylene glycol) copolymer (PDMS-PEG) into the PDMS cure when fabricating the mold.^39,40^ This significantly reduced the mold surface hydrophobicity, as determined by water contact angle measurements (Table 1 and Figure S1A–C).

**Table 1:** Water Contact Angles on PDMS Containing Varying Amounts of PDMS-PEG, of Water Droplets Containing Tween-20, and on lawns of MS^a^

0% PDMS-PEG	0.1% PDMS-PEG	1% PDMS-PEG	0.1% Tween-20	ENG-MS	PS-MS	G-PS-MS
105 ± 4°	84 ± 4°^b^	64 ± 11°^b^	40 ± 5°^b^	130 ± 4°	127 ± 7°	115 ± 7°^c^

a Data represent averages ± standard deviation of *n* = 8 replicates.

b Significantly different from 0% PDMS-PEG, Student’s *t*-test, *p* < 0.00005.

c Significantly different from PS-MS, Student’s *t*-test, *p* < 0.01.

Second, we supplemented the casting solution with 0.1% (v/v)Tween-20, which is a nonionic, biocompatible surfactant that is often used as a dispersant in microsphere suspensions.^41^ The addition of Tween-20 significantly reduced water contact angle on PDMS (Table 1 and Figure S1D).

Third, we reduced the hydrophobicity of MSs using polystyrene MSs (PS-MSs) coated with G protein (G-PS-MSs) and found that G protein coating significantly reduced MS hydrophobicity compared to uncoated PS-MSs (Table 1 and Figure S1F,G). We used the commercially available PS-MSs because formulating PLGA-ENG-MS to have a G protein coating would have required significant technical effort and would have produced MSs with no apparent future use other than for this individual experiment. We believe that this substitution is appropriate because ENG-MSs and uncoated PS-MSs have similar hydrophobicity (Table 1 and Figure S1F,G).

#### 2.2.2 Effect of PDMS with Reduced Hydrophobicity and Addition of Tween-20 Surfactant on MS Loading in MN Tips

**Figure 3:**
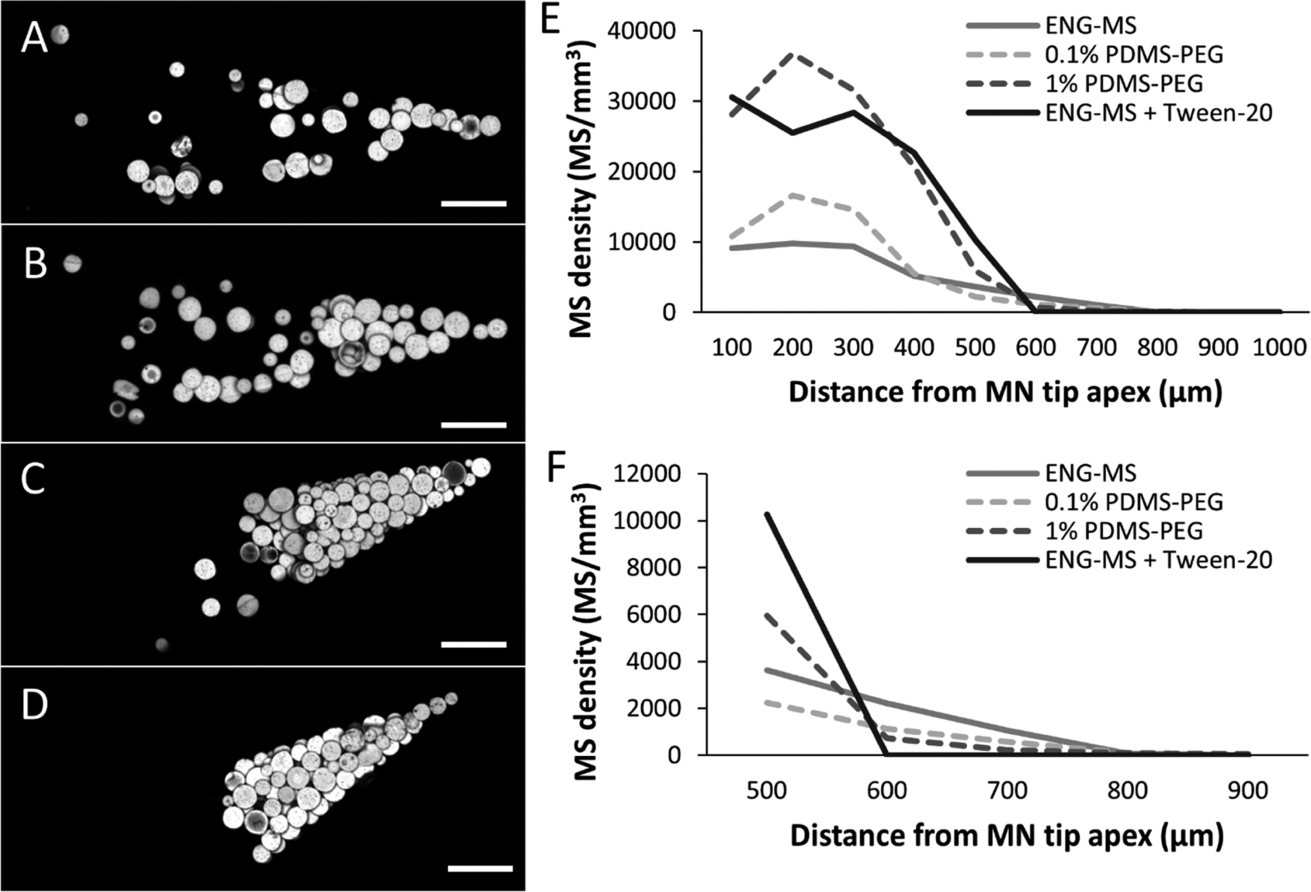
Effect of mold hydrophobicity and surfactant on ENG-MS distribution in MNs. Representative confocal microscopy images of individual MNs containing ENG-MSs cast without surfactant into PDMS molds containing (A) 0% PEG, (B) 0.1% PEG, or (C) 1% PEG, or cast (D) with 0.1% v/v Tween-20 into a PDMS mold containing no PEG. Scale bars are 100 μm. (E) ENG-MS density in volumes of 100 μm distance increments from the MN tip apex. (F) Expanded view of data shown in (E). The MN casting solution comprised 6% w/v PVP + 6% w/v sucrose in water with 0.1% w/v rhodamine 6G-labeled ENG-MSs (and 0.1% v/v Tween-20 when indicated). The backing casting solution comprised 18% w/v PVP + 18% w/v sucrose in water. ENG-MSs had a diameter of 37 μm. Data represent the mean of *n* = 4 replicates.

We imaged ENG-MS distribution in MNs by confocal microscopy after casting onto MN molds with varied hydrophobicities and found that ENG-MS localization in MN tips increased as hydrophobic forces were weakened (Figure 3A–C).

When plotting the density of ENG-MSs as a function of distance from the MN tip apex, we observed three different trends (Figure 3E,F). First, the reduced hydrophobicity of the mold (i.e., more PDMS-PEG content) correlated to denser ENG-MS packing in the MN tips that reached a maximum of ∼30,000 MS/mm^3^. Second, the reduced hydrophobicity of the mold also correlated with a larger packed tip, characterized by the distance from the tip apex where the ENG-MS density rapidly dropped off. Third, the reduced hydrophobicity of the mold correlated with lower densities of ENG-MS farther outside the tip, characterized by the smaller and shorter tails for the 0 and 0.1% PDMS-PEG density plots compared to the 1% PDMS-PEG plot (Figure 3F). Altogether, these data support the hypothesis that reducing the hydrophobic interactions that cause ENG-MSs to adhere to the PDMS mold walls can increase ENG-MS loading into MN tips.

We also considered using plasma treatment to increase the mold surface hydrophilicity, but did not pursue this approach because the effects of plasma treatment alone were short-lived, and we found that use of plasma treatment to bind poly(vinyl alcohol) (PVA) to the mold surface as a longer-term solution led to the formation of MNs that could not be removed from the mold (data not shown).

We next assessed the impact of reducing attractive hydrophobic interactions by adding Tween-20 surfactant to the casting solution. This intervention also led to increased packing of ENG-MSs in MN tips (Figure 3D) and generated an ENG-MS density plot with increased MS packing in large MN tips with no tail of MSs outside the tip (Figure 3E,F). The effects of surfactant on MS distribution in the MNs was similar to that of the least-hydrophobic PDMS-PEG mold and appeared to provide greater MN tip localization than the more-hydrophobic molds.

**Figure 4:**
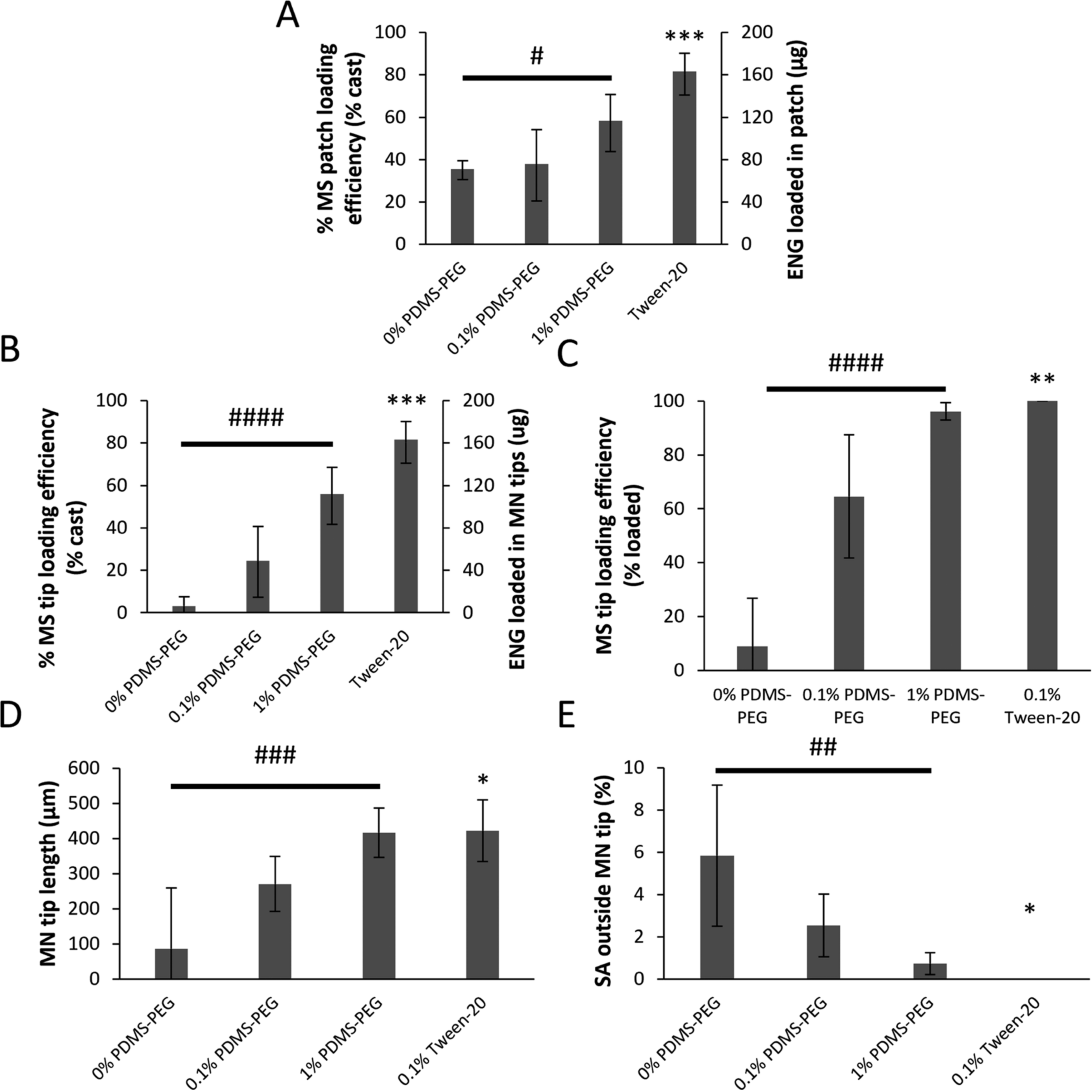
Quantitative effects of mold hydrophobicity and surfactant on ENG-MS distribution in MNs. (A) Amount of ENG loaded per MN patch and loading efficiency of ENG-MSs in a MN patch expressed as a percentage of the total ENG-MSs used in the manufacturing process. (B) Amount of ENG loaded in the tips of a MN patch and loading efficiency of ENG-MSs in the tips of a MN patch expressed as a percentage of the total ENG-MSs used in the manufacturing process. (C) Loading efficiency of ENG-MSs in the tips of a MN patch expressed as a percentage of the total ENG-MSs loaded into the MN patch. (D) Length of MN tip (i.e., region of MN densely packed with ENG-MSs). (E) Percentage of the surface area of MN outside of tip covered by ENG-MSs. The MN casting solution comprised 6% w/v PVP + 6% w/v sucrose in water with 0.1% w/v rhodamine 6G-labeled ENG-MSs (and 0.1% v/v Tween-20 when indicated). The backing casting solution comprised 18% w/v PVP + 18% w/v sucrose in water. ENG-MSs had a diameter of 37 μm. Student’s *t*-test compared to 0% PDMS-PEG: *p < 0.05; **p < 0.005; and ***p < 0.0001. ANOVA: ^#^*p* < 0.05; ^##^*p* < 0.005; ^###^*p* < 0.0005; and ^####^*p* < 0.00001. Data represent mean ± standard deviation of *n* = 4 replicates.

We quantified the differences in ENG-MS loading among these four ENG-MS loading scenarios by counting ENG-MSs by microscopy (e.g., Figure 3A–D) and by high-performance liquid chromatography (HPLC) quantification of ENG and evaluated these findings by five different metrics. Our first assessment was of MSs and ENG content in MN patches (i.e., without considering distribution within the MN patches or localization to the MN tips). This analysis assessed the amount and efficiency of ENG used in the manufacturing process that would be incorporated into the MN patches. We found significantly higher MS patch loading efficiency and ENG patch loading when using the PDMS-PEG molds and when casting using a casting solution formulation containing Tween-20 surfactant compared to the control scenario of casting without Tween-20 onto PDMS molds without PEG (Figure 4A). In these data, the MS patch loading efficiency and the ENG patch loading directly scale with each other because the ENG content per MS (i.e., 27% w/w), and the total amount of MS cast per mold (i.e., 196 μg) was held constant. These findings suggest that mitigating hydrophobic interactions not only allows ENG-MSs to more easily slide down the MN mold cavity walls to fill MN tips but it may also help prevent adhesion of the MSs on the upper surface of the PDMS mold and into the MN mold cavities, thereby improving overall loading.

We next quantified the efficiency of ENG-MS loading in the MN tips as opposed to other parts of the MN patches, as a measure of the amount and efficiency of ENG used in the manufacturing process that would be expected to be delivered into skin from the MN tips. Here, we defined “tips” as the portion of the MN with densely packed MSs as opposed to other parts of the MN with MSs dispersed at low density. This analysis revealed greater MS tip loading efficiency and greater ENG loading in MN tips when using the PDMS-PEG molds and the Tween-20 formulation compared to control (Figure 4B). Using Tween-20, we were able to load ∼80% of the ENG-MS into the MN tips, which compared to just ∼3% of ENG-MSs loaded into the MN tips without the surfactant or hydrophilic molds.

As a third measure of loading efficiency, we determined MN tip loading as a percentage of ENG-MSs loaded into the MN patch, which is a more commonly used measure of expected MN patch delivery efficiency found in the literature.^42^ We again found significantly higher MS tip loading efficiency when using less-hydrophobic molds or Tween surfactant (Figure 4C). The 1% PDMS-PEG molds and casting with Tween-20 both yielded close to 100% MS tip loading efficiency by this measure. A fourth way to characterize MN tip loading is by the length of the densely packed MN tips, which were 3–5-fold longer when using the less-hydrophobic molds and the Tween surfactant compared to control (Figure 4D). Finally, we quantified the surface area outside the MN tips covered by ENG-MSs as a measure of how spread out the nonlocalized MSs were and found much less ENG-MSs covering the MN outside of the tip when using the molds with PDMS-PEG or the casting formulation with Tween-20 compared to control (Figure 4E).

#### 2.2.3 Effect of MSs with Reduced Hydrophobicity on MS Loading in MN Tips

We also varied the MS hydrophobicity to influence interactions between MSs and PDMS walls by comparing MN tip loading of PS-MSs to G-PS-MSs (Figure S2). While MNs loaded with G-PS-MSs trended toward improved tip loading, differences compared to PS-MS loading were not significant (Figures S2 and S3). This may be explained by the relatively small reduction in hydrophobicity associated with the G protein coating (Table 1).

### 2.3 Effect of Electrostatic Interactions on MS Localization

In addition to hydrophobicity, we also investigated the effect of electrostatic interactions on the localization of MS in MN tips. For this study, we again used MSs made of polystyrene but with a smaller diameter (7 μm) (sPS-MSs) compared to the PS-MSs used above (31 μm). We originally selected the smaller-sized sPS-MSs because there were available with additional functionality that we thought would be useful but later found not to be useful.

Like PLGA and PDMS, PS is typically negatively charged due to the presence of a negatively charged initiator on the surface of the MSs after polymerization.^43,44^ As a result, we expect electrostatic repulsion between the MSs and the PDMS mold to aid in sPS-MS localization, and this effect was present in the above experiments assessing hydrophobicity. We therefore sought to isolate the effects of electrostatic interactions and put them in context with the observed effects of hydrophobicity.

**Figure 5:**
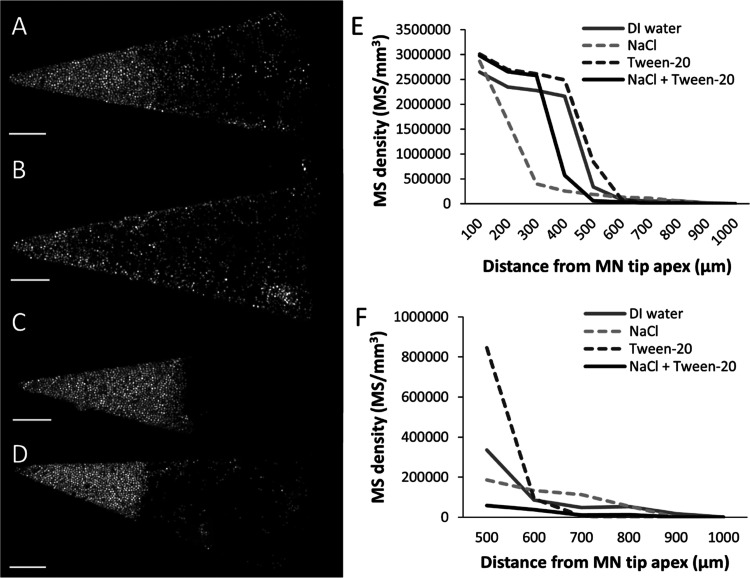
Effect of electrostatic forces on sPS-MS distribution in MNs. Representative confocal microscopy images of individual MNs containing sPS-MSs cast in DI water with (A) no excipients, (B) 1 M NaCl, (C) 0.1% v/v Tween-20, or (D) 1 M NaCl + 0.1% v/v Tween-20. Scale bars are 100 μm. (E) sPS-MS density in volumes of 100 μm distance increments from the tip. (F) Expanded view of data shown in (E). The MN casting solution comprised 0.1% w/v yellow fluorescence-labeled sPS-MSs in DI water (and 0.1% v/v Tween-20 or 1 M NaCl when indicated). The backing casting solution comprised 18% PVP + 18% sucrose in water. sPS-MSs had a diameter of 7 μm. Data represent the mean of *n* = 4 replicates.

To minimize electrostatic interactions, we added 1 M NaCl to the casting solution with the goal of screening electrostatic interactions between sPS-MSs and the PDMS mold walls (i.e., 1 M NaCl is expected to reduce Debye length to ∼0.3 nm^34^). Compared to casting sPS-MSs in deionized (DI) water, which formed a MN tip of densely packed MSs (Figure 5A), the addition of NaCl to the casting solution resulted in sPS-MSs distributed throughout the MN that failed to form a dense MN tip (Figure 5B). This indicates that electrostatic repulsion played a role in preventing sPS-MSs from adhering to the mold walls and that screening this repulsive force with salt increased sPS-MS adhesion to the mold walls.

As expected, addition of Tween-20 to the sPS-MS casting solution (without NaCl) led to good MN tip formation (Figure 5C). Finally, when we prepared a casting formulation containing both NaCl and Tween-20, which is expected to lack electrostatic repulsion (due to NaCl) and minimize hydrophobic attraction (due to Tween-20) between sPS-MSs and PDMS, we formed densely packed MN tips (Figure 5D). This indicates that blocking attractive hydrophobic forces with the surfactant was sufficient to enable MN tip formation, even in the absence of repulsive electrostatic forces screened by NaCl. However, the sPS-MS density distribution data suggest that MN tip size was bigger when repulsive electrostatic interactions were present to further overcome possible attractive forces between sPS-MSs and the PDMS walls (Figure 5E).

**Figure 6:**
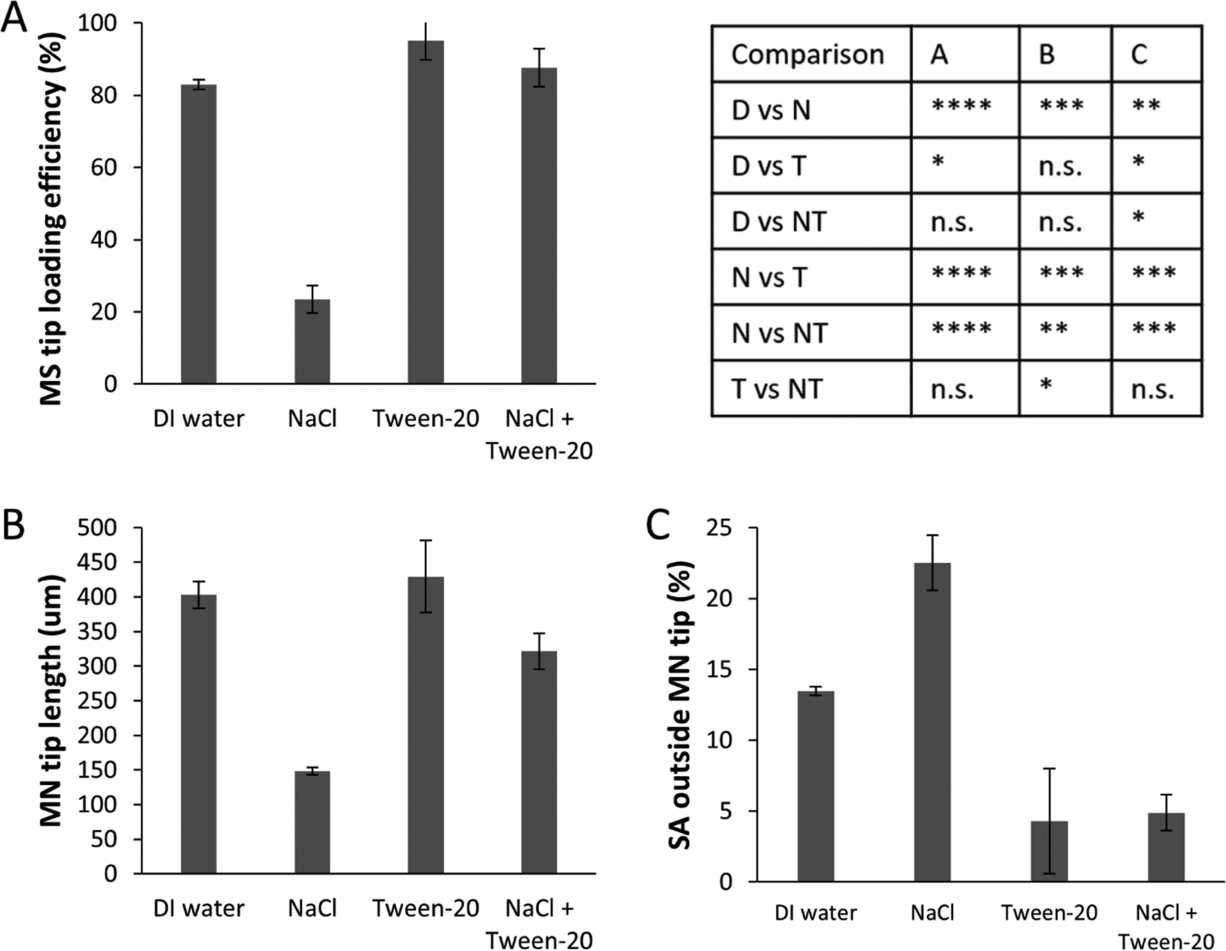
Quantitative effects of electrostatic forces on sPS-MS distribution in MNs. (A) Loading efficiency of sPS-MSs in the tips of a MN patch expressed as a percentage of the total PS-MSs loaded into the MN patch. (B) Length of MN tip. (C) Percentage of the surface area of MN outside of tip covered by sPS-MSs. The MN casting solution comprised 0.1% w/v yellow fluorescence-labeled sPS-MSs in DI water (and 0.1% v/v Tween-20 or 1 M NaCl when indicated). The backing casting solution comprised 18% w/v PVP + 18% w/v sucrose in water. sPS-MSs had a diameter of 7 μm. The significance of the statistical comparison is shown in the table. D = DI water, T = Tween-20, N = NaCl, and NT = NaCl + Tween-20. One-way ANOVA with Tukey–Kramer post hoc test: *p < 0.05; **p < 0.0005; ***p < 0.00005; ****p < 0.00000001; and n.s. *p* > 0.05. Data represent mean ± standard deviation of *n* = 4 replicates.

Additional quantitative analysis of sPS-MS distribution in MN tips further reinforced the conclusion that electrostatic repulsion can play an important role in reducing MS interactions with the PDMS mold and thereby promote the formation of densely packed MN tips. Measures of tip loading efficiency of sPS-MSs (Figure 6A), MN tip length (Figure 6B), and surface area covered by MSs outside the MN tip (Figure 6C) all showed worse MN tip formation when NaCl was added to screen repulsive electrostatic forces. Addition of the surfactant to minimize attractive hydrophobic forces was sufficient to significantly promote MN tip formation, even in the absence of repulsive electrostatic forces screened by NaCl (Figure 6A–C), which again shows that electrostatic repulsion may help but is not needed for MN tip formation as long as hydrophobic attractive forces are blocked.

### 2.4 Effect of Optimized MN Patches on ENG Delivery to Skin

**Figure 7:**
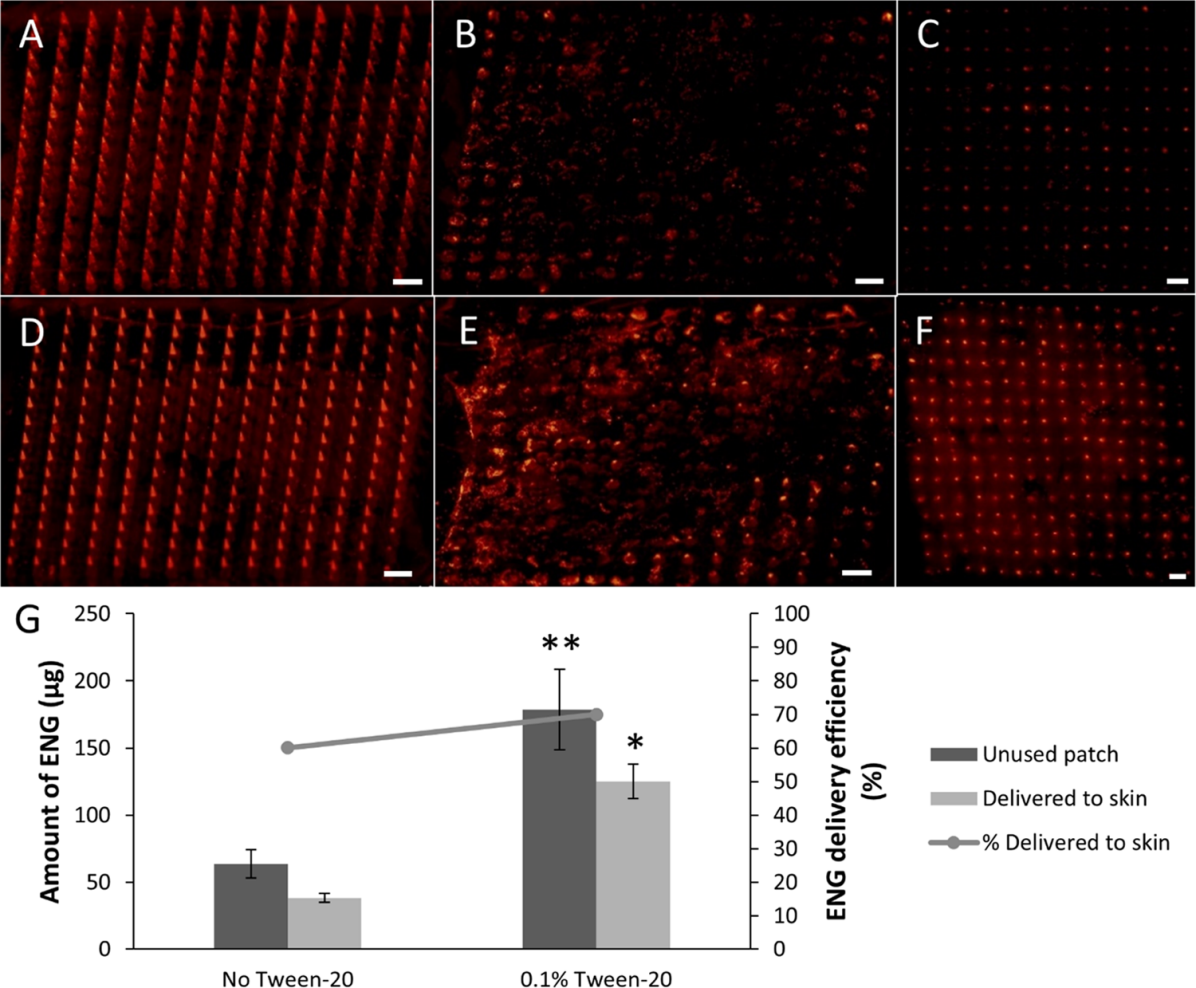
Delivery of ENG-MSs from MN patches into pig skin ex vivo. Representative fluorescence microscopy images of (A) an unused MN patch, (B) a used MN patch, and (C) pig skin after application of an MN patch that was formulated without Tween-20. Representative fluorescence microscopy images of (D) an unused MN patch, (E) a used MN patch, and (F) pig skin after application of an MN patch that was formulated with Tween-20. Scale bar for all images is 1 mm. (G) Quantification of ENG in unused patches and ENG delivered into pig skin (bars), as well as the percentage of ENG in unused patches that was delivered into pig skin (line). The MN casting solution comprised 6% w/v PVP + 6% w/v sucrose with 0.1% w/v rhodamine 6G-labeled MS in water (and 0.1% v/v Tween-20 when indicated). The backing casting solution comprised 18% w/v PVP + 18% w/v sucrose in water. Student’s *t*-test, compared to no Tween-20: *p < 0.05; **p < 0.005. Data represent mean ± standard deviation of *n* = 3 replicates.

To assess the delivery of MSs to the skin using optimized MN patches, we applied MN patches made with and without Tween-20 for delivery of negatively charged ENG-MSs to porcine skin ex vivo (Figure 7A,D).

We found that substantial MN dissolution and ENG-MS delivery occurred after application to the skin (Figure 7B,E). Examination of the skin demonstrated deposition of ENG-MSs in the skin, with greater MS delivery by the Tween-20-containing MN patches (Figure 7F) shown by the greater fluorescence compared to the MN patches without the surfactant (Figure 7C).

Quantification of ENG in unused and used patches immediately after patch application revealed that MNs without Tween-20 were loaded with 64 ± 11 μg of ENG and delivered 38 ± 4 μg of ENG into skin, while MNs with Tween-20 were loaded with 179 ± 30 μg of ENG and delivered 125 ± 13 μg of ENG (Figure 7G). This represents an almost 3-fold greater ENG loading and a more-than 3-fold greater ENG delivery to the skin. The ENG delivery efficiency into skin was 60 ± 6% without Tween-20 and 70 ± 7% with Tween-20, which were not significantly different (Student’s *t*-test, *p* = 0.13, Figure 7G).

## 3 Conclusions

MS-loaded MNs present an exciting application of MN patches that combines well-established controlled release MS technology with a self-administrable MN patch drug delivery platform. This study investigated challenges associated with the fabrication of such MS-loaded MN patches.

First, we showed that MSs in suspension can fail to localize in the MN tip (which can impede MS delivery into skin) and found that MSs can adhere to the PDMS mold walls during MN patch fabrication.

Next, we demonstrated that hydrophobic interactions were a major cause of this adhesion by showing improved MS localization in the tips when we decreased mold, casting solution, and MS hydrophobicity. We also showed that repulsive electrostatic interactions between MSs and the mold aided MS localization in the tips, though hydrophobic interactions were more significant.

Finally, we showed 3-fold greater ENG loading in the MN patches and delivery into skin when MN patches were fabricated to reduce hydrophobic interactions with Tween-20 than without. We conclude from these data that interventions targeting the hydrophobic interactions can significantly enhance MS loading in MN patches, improve localization in MN tips, and increase both delivered dose and delivery efficiency in the skin. These findings may further address MS delivery challenges in other contexts,^45,46^ such as MS injection using traditional hypodermic syringes, where MSs are also found to stick to the vial, syringe, and needle walls, which can reduce dosing.^47−49^

## 4 Experimental Section

### 4.1 MS-loaded MN Fabrication

PLGA-ENG-MSs were fabricated by a solid-oil-water emulsion/solvent evaporation method. Briefly, 137 mg of ENG was added into a solution of PLGA (320 mg, Resomer RG 503 H, Sigma-Aldrich, St. Louis, MO, acid terminated, LA:GA = 50:50, MW 24–38 kDa) in 1 mL of methylene chloride and then homogenized using Tempest IQ2 homogenizer (VirTis Company, Gardiner, NY) at 10,000 rpm for 2 min. Four milliliters of 5% PVA solution was added to the above mixture and vortexed on a Vortex-Genie 2 shaker (Scientific Industries lnc., Bohemia, NY) for 1 min to form a S/O/W emulsion, which was immediately transferred into 100 mL of 0.5% PVA solution and stirred with an IKA EUROSTAR 20 Digital Overhead Stirrer (IKA Works, Staufen, Germany) at 700 rpm at room temperature for 3 h for solvent evaporation. Microspheres were sieved (20–45 μm), washed, and then collected by centrifugation at 4000 rpm for 5 min. The microspheres were lyophilized for 48 h under reduced pressure and stored at 4 °C before use. Fluorescent dye-labeled PLGA MSs were prepared by adding 0.4% (w/v) rhodamine 6G into the ENG and PLGA solution in methylene chloride at the beginning of the fabrication process.

Yellow-fluorescent polystyrene MSs (sPS-MSs, 7 μm diameter) were obtained from Magsphere (Pasadena, CA). Uncoated (PS-MSs, 31 ± 0.7 μm diameter) and G protein-coated (G-PS-MSs, 41 ± 3.0 μm diameter) yellow-fluorescent (peak excitation/emission of 480:469–553 nm) polystyrene MSs were obtained from Spherotech (Lake Forest, IL). For these commercially purchased MSs, suspension excipients such as surfactants were removed by diluting the suspension in excess DI water, centrifuging at 3000*g* (Thermo Scientific, Waltham, MA), removing the supernatant, and repeating 3–5 times. The resulting MS suspension was then lyophilized (SP Scientific, Gardiner, NY), and the dry powder form was stored with desiccant until use.

MN molds were prepared by casting PDMS (Sylgard 184, Dow, Midland, MI) onto MN master structures in the shape of MN arrays made of poly(lactic acid), as described previously.^50^ To vary PDMS mold hydrophobicity, 0.1 and 1% w/w PDMS-PEG (60–70% PEG) block copolymer (Gelest, Morrisville, PA) was mixed into the precured PDMS and cured, as previously described.^39,40^ Modified PDMS molds were then rinsed in DI water overnight.

MSs were dispersed as a suspension in casting solution with an ultrasonic bath (Fisher Scientific, Hampton, NH) for 30 min. Solution excipients included poly(vinylpyrrolidone) (PVP) and sucrose at 6% w/v each, Tween-20 at 0.1% w/v, and/or NaCl at 1 M concentrations (all from Sigma-Aldrich). Up to 200 μL of the casting suspension was first applied to the top of a PDMS mold under vacuum for 15 min (without centrifugation) before capping with a PDMS cap and centrifuging at 3000*g* for an additional 15 min.

The excess suspension was then scraped off, and the casting process was repeated three more times. Finally, the PDMS mold was topped with up to 400 μL of a backing solution composed of 18% w/v PVP and 18% w/v sucrose under vacuum for up to 3 h. After casting, MNs were dried at 40 °C on a hot plate (Torrey Pines Scientific, Carlsbad, CA) overnight before gentle removal from the mold by peeling with tape and stored in a desiccator at room temperature (20–25 °C) until use.

### 4.2 MN and Mold Characterization

To prepare MNs for confocal microscopy imaging, half of the MNs (i.e., those on the periphery of the patch) were carefully scraped off by a razor blade to access MNs in the center of the patch. Individual MNs were then carefully removed by a razor blade and laid horizontally on a glass microscope cover slip. A drop of oil (Immersol, Zeiss, Oberkochen, Germany) was then applied on top of the MN, and the MN was imaged with a confocal microscope (LSM 900, Zeiss) focused halfway through its thickness. All MSs were excited with 488 nm wavelength laser. ENG-MS emission was detected at 521–800 nm, while PS-MS emissions were detected at 400–650 nm.

Images captured by confocal microscopy were processed with ImageJ (National Institutes of Health, Bethesda, MD). The length scale was first calibrated with the scale bar. The MN was then characterized in terms of the existence and dimensions (length, base diameter) of a packed tip of MSs and the density distribution of the MS in the MN in 100 μm increments from the apex of the MN tip. A packed tip was defined as a region of the MN starting from the apex of the MN tip that was densely filled with MS. The density distribution of MSs in MNs was calculated as described in the Supporting Information.

Loading of ENG in MN patches was determined by HPLC. ENG separation was achieved with an Eclipse XDC-C18 column (Agilent, Santa Clara, CA) at 50 °C. The mobile phase was an 80:20 v/v mixture of acetonitrile and DI water, the flow rate was 1 mL/min for 5 min, and UV absorbance was measured at 245 nm. First, both unused and used MN patches containing ENG-MSs were each dissolved in 5 mL of DI water for at least 15 min. The suspension was subsequently centrifuged at 3000*g* for 15 min, after which 4 mL of the supernatant was carefully removed and replaced by 4 mL of acetonitrile (Sigma-Aldrich). The ENG was allowed to extract into the solution for 30 min before the solution was centrifuged at 3000*g* for 15 min. Finally, 500 μL of solution was added to 500 μL of acetonitrile, and the resultant 10× dilution was quantified by HPLC. A standard curve was also generated with ENG dissolved in acetonitrile at concentrations of 100, 50, 25, 10, and 5 μg/mL.

The hydrophobicity values of MS and PDMS molds were determined by water contact angle. To prepare the MSs for water contact angle measurement, 50 mg of ENG-MSs, PS-MSs, or G-PS-MSs was suspended in 3 mL of DI water and injected onto a piece of 10 μm filter paper made of polyethersulfone (Millipore, Burlington, MA) to yield a uniform lawn of MSs on top of the filter paper. To prepare the PDMS molds for water contact angle measurement, the molds were cleaned with a tape strip. Briefly, 10 μL of DI water was carefully dropped onto the mold surface and allowed to equilibrate for 10 min. Images of the water droplet were then taken by phone camera (S10, Samsung, Suwon, South Korea) and the water contact angle was determined by ImageJ.

### 4.3 Ex Vivo Skin Insertion

MN patch insertion was evaluated in ex vivo porcine skin. Excised porcine skin (obtained from a slaughterhouse and stored frozen until use) was separated from the subcutaneous fat, shaved, cleaned with an isopropyl alcohol wipe, stretched and pinned on top of a moist Kimwipe, and allowed to equilibrate for ∼30 min. The MN patches were then manually pressed onto the skin for 30 s with a 15 lb force and left on the skin for 15 min before removal. Scotch tape was then used to strip away any residual MS left on the skin.

The quantity of ENG delivered into skin was calculated by subtracting the average amounts of ENG in a used patch and left as residual on the skin surface (and collected by tape strip) from the average amount of ENG in an unused patch. The ENG delivery efficiency was calculated by dividing the average amount of ENG delivered by the average amount of ENG in an unused patch.

### 4.4 Statistical Analysis

All results in this study are presented as mean ± standard deviation. Statistical analysis was performed using Excel (Microsoft, Redmond, WA). Two-sided Student’s t-test and one-way ANOVA with Tukey–Kramer’s post hoc test were used, and comparisons with *p* < 0.05 were considered statistically significant.

## Supporting Information Available

Additional experimental methods for calculation of MS density distributions and choice of MSs. Data on water contact angle images (Figure S1); PS-MS density distribution in MNs (Figure S2); and quantitative effects of hydrophilicity on PS-MS distribution (Figure S3) (PDF)

## Abbreviations

DI: deionized
ENG: etonogestrel
ENG-MS: etonogestrel-loaded microsphere
G-PS-MS: G protein-coated polystyrene microsphere
MN: microneedle
MS: microsphere
PDMS: poly(dimethylsiloxane)
PDMS-PEG: PDMS-poly(ethylene glycol)
PLA: poly(lactic acid)
PLGA: poly(lactic-*co*-glycolic acid)
PS-MS: polystyrene microsphere
PVP: poly(vinylpyrrolidone)
sPS-MS: small polystyrene microsphere
