# Supplementary material for: Effect of Surface Interactions on Microsphere Loading in Dissolving Microneedle Patches
Source: ACS Appl Mater Interfaces. 2022 Jun 22;14(26):29577–87. doi: 10.1021/acsami.2c05795 (PMC9264316; doi:10.1021/acsami.2c05795)
Supplement: Supplementary file 1 — am2c05795_si_001.pdf [file am2c05795_si_001.pdf]

Supporting Information

Effect of Surface Interactions on Microsphere Loading in Dissolving Microneedle Patches

Derek Jang*, Jie Tang†, Steven P. Schwendeman†, and Mark R. Prausnitz*‡

*Wallace H. Coulter Department of Biomedical Engineering at Georgia Tech and Emory University, Georgia Institute of Technology, Atlanta, Georgia 30332, United States

†Department of Pharmaceutical Sciences and the Biointerfaces Institute, University of Michigan, Ann Arbor, Michigan 48109, United States

‡School of Chemical & Biomolecular Engineering, Georgia Institute of Technology, Atlanta, Georgia 30332, United States

Corresponding Author

Mark R. Prausnitz – School of Chemical and Biomolecular Engineering, Georgia Institute of Technology, Atlanta, Georgia 30332, United States; orcid.org/0000-0002-9076-8448; Email: prausnitz@gatech.edu.

Supplementary Experimental Methods

MS density distribution

The density distribution of the MS was divided into 3 categories for analysis: "tip of the tip", "tip", and "outside the tip". MS were considered to be in the "tip of the tip" if they were completely countable within the tip (i.e. there were no MS hidden from view by being blocked by other MS). The remaining MS in the tip were treated separately as "tip", and MS outside the tip were considered "outside the tip."

For ENG-MS and PS-MS (i.e., with diameter of 30 – 40 µm), each MS was individually counted and categorized into one of the three categories. For sPS-MS (i.e., with 7 µm diameter), an average density of MS in the tip was determined by drawing a shape in ImageJ, calculating its area, and counting the number of MS in the area. This was repeated five times and the number of MS in the tip was determined by multiplying the average density by the image area of each 100 µm increment, given by 100×0.5×(R+r) μm^2 where r is the top radius and R is the bottom radius for each increment. This method was used for the sPS-MS because they were too numerous to be manually countable in the tip. MS in the "tip of the tip" and "outside the tip" were individually counted.

"Tip of the tip" MS were fully countable by eye and did not need additional calculations to determine the number of MS. We assumed a symmetric distribution of MS in the MN so the number of MS "outside the tip" was doubled to account for the other half of the MN that was not imaged. To determine the number of MS within the "tip", we accounted for MS hidden behind the visible layer of MS by making several assumptions. First, we assumed an average MS diameter (d) as given by the MS manufacturer. We then assumed that the visible MS in the tip (MS_s) accounted for a truncated cone shell volume with thickness equal to the average MS diameter; as a result, we needed to calculate the number of MS in the remaining hidden volume. For every 100 µm increment, the inner truncated cone therefore had a top radius of (r-d) and bottom radius of (R-d), giving it a volume of $\boldsymbol{V_i = \frac{1}{3} \times \pi \times h \times \{(r-d)^2 + (r-d)(R-d) + (R-d)^2\}}$.

The truncated shell therefore had a volume of $\boldsymbol{V_s = \frac{1}{3} \times \pi \times h \times (r^2 + rR + R^2) - V_i}$. The number of MS in V_i (MS_i) was calculated by $\boldsymbol{MS_i = 2 \times MS_s \times \frac{V_i}{V_s}}$, where MS_s was doubled to account for the half of the MN that was not imaged. The total number of MS in the tip was therefore the sum of MS_s and MS_i.
The density distribution of MS was determined by summing the total number of MS in each 100 µm increment divided by the increment volume V, where $\boldsymbol{V = \frac{1}{3} \times \pi \times h \times (r^2 + rR + R^2)}$. To calculate the percentage of MS in the tip, the sum of "tip of the tip" and "tip" MS was divided by the total number of MS in the MN. To calculate the percentage of surface area outside the tip that was covered by MS, the surface area was first determined by the formula $\boldsymbol{SA = \pi \times (r+R) \times \sqrt{(R-r)^2 + h^2}}$. The surface area covered by MS (SA_m) was the number of MS multiplied by $\boldsymbol{\pi\left(\frac{d}{2}\right)^2}$, giving the percentage of surface area outside the tip that was covered by MS as $\boldsymbol{\frac{SA_m}{SA}}$.

To normalize the density distributions of the PS-MS with different diameters, the densities of G-PS-MS were multiplied by $\boldsymbol{\frac{PS-MS_r{}^3}{G-PS-MS_r{}^3}}$, where $PS\text{-}MS_r$ and $G\text{-}PS\text{-}MS_r$ are the radii of PS-MS and G-PS-MS, respectively.

Supporting Figures

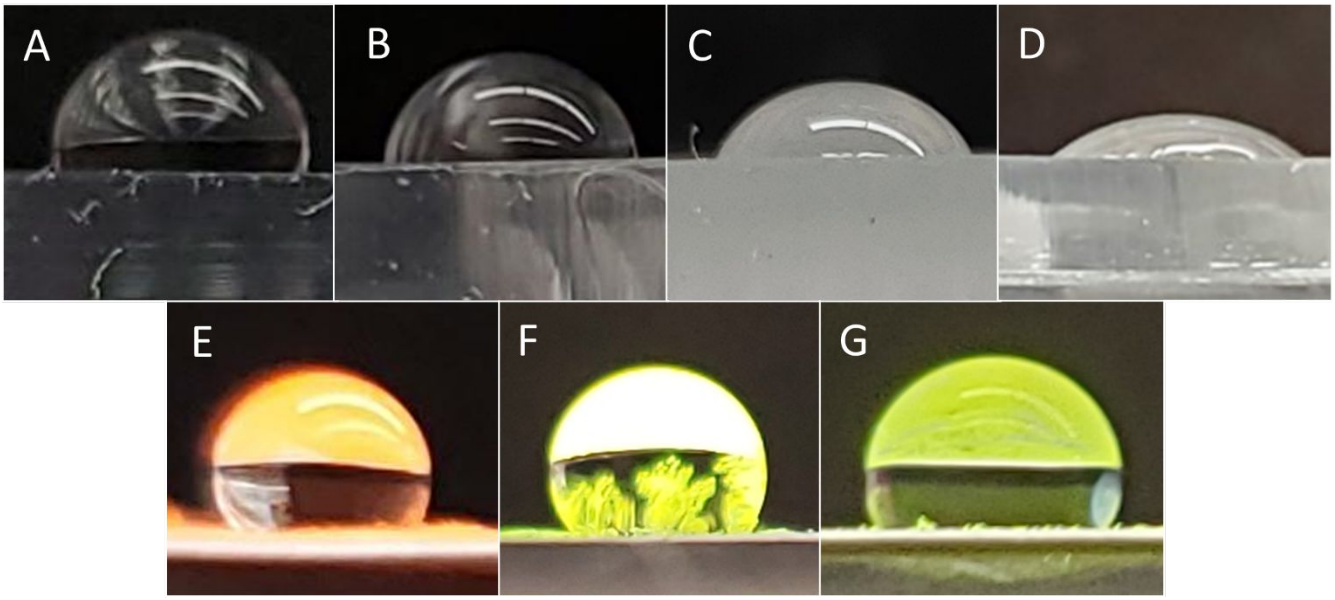

Figure S.1 Contact angle of water droplets on PDMS and MS surfaces. Representative images of 10 µl water droplets on a PDMS mold containing (A) 0%, (B) 0.1% w/w, or (C) 1% w/w PDMS-PEG, (D) with 0.1% v/v Tween-20 on a PDMS mold containing 0% PDMS-PEG, or on a lawn of (E) ENG-MS, (F) uncoated PS-MS or (G) G protein-coated PS-MS.

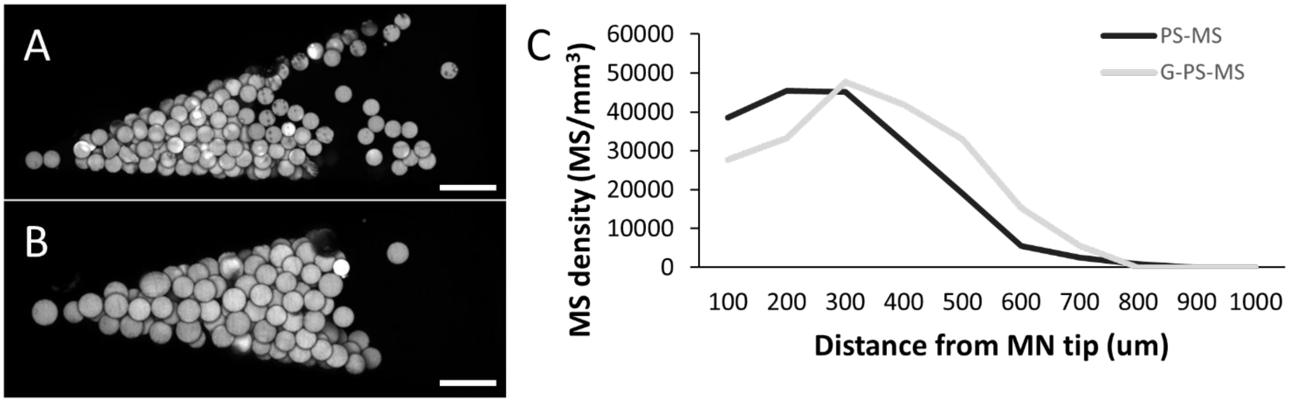

Figure S.2 Effect of MS hydrophobicity on PS-MS distribution in MNs. Representative confocal microscopy images of individual MNs containing PS-MS with and without G protein coating to decrease surface hydrophobicity. Scale bars are 100 µm. (C) PS-MS density in volumes of 100 µm distance increments from the tip. G-PS-MS density was normalized to PS-MS density based on their different MS diameters (see Methods). The MN casting solution comprised 0.1% w/v yellow fluorescence-labeled PS-MS in DI water. The backing casting solution comprised 18% w/v PVP + 18% w/v sucrose in water. PS-MS had a diameter of 31 µm and G-PS-MS had a diameter of 41 µm. Data represent mean of n = 4 replicates.

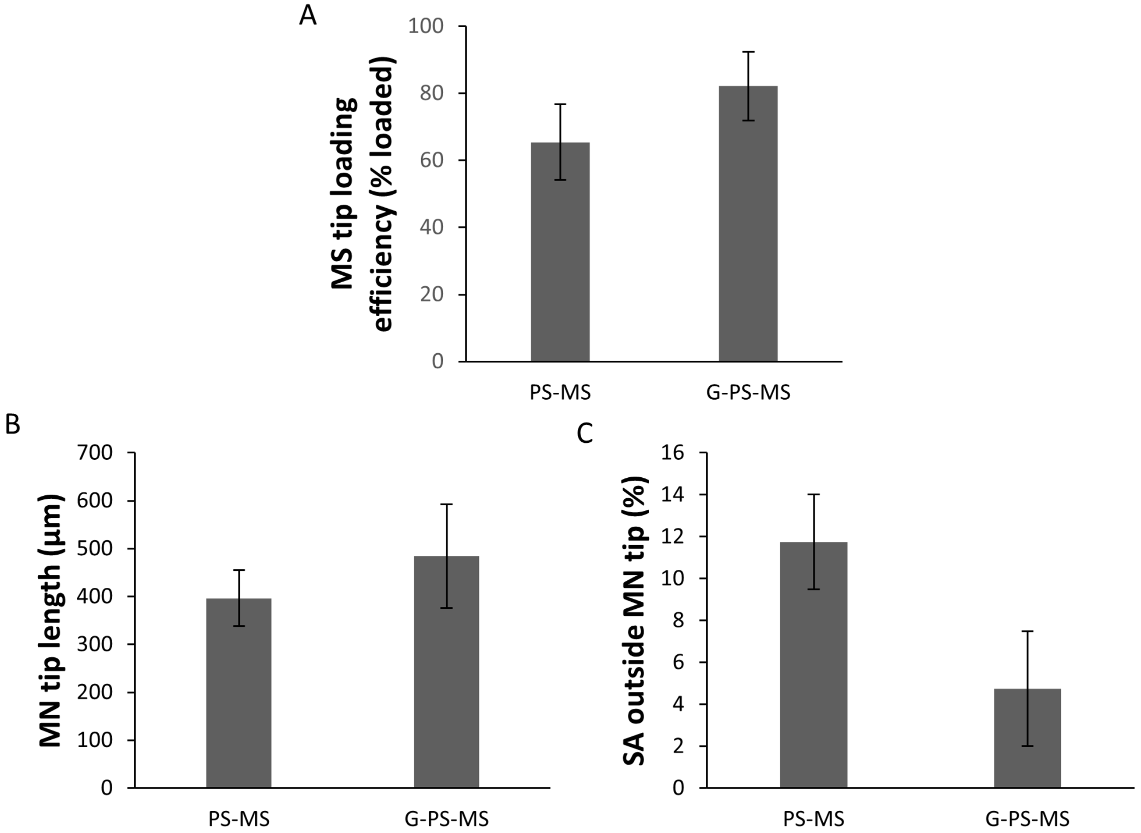

Figure S.3 Quantitative effects of MS hydrophilicity on PS-MS distribution in MNs. (A) Loading efficiency of PS-MS in the tips of a MN patch expressed as a percentage of the total PS-MS loaded into the MN patch. (B) Length of MN tip. (C) Percentage of surface area of MN outside of tip covered by PS-MS. The MN casting solution comprised 0.1% w/v yellow fluorescence-labeled PS-MS in DI water. The backing casting solution comprised 18% w/v PVP + 18% w/v sucrose in water. PS-MS had a diameter of 31 µm and G-PS-MS had a diameter of 41 µm. Data represent mean ± standard deviation of n = 4 replicates.
